# Inhalation of Sulphuric Ether—Its Danger

**Published:** 1847-05

**Authors:** 


*Inhalation of Sulphuric Ether—Its danger.*—Already do we begin to hear of dangers and accidents in the use of this patent narcotic, and as as a faithful annalist we are bound to record them. It has been tried in an Insane Asylum in Paris, and its "effects were unsatisfactory and even dangerous."

"In an instance too, mentioned in the same letter by Dr. Fisher, where it was inhaled by a parturient woman, the eyes of the patient were injected to such a degree that the blood seemed almost to gush out from them, the tongue became swollen and a frothy saliva flowed freely from the mouth—a sudden and alarming cerebral congestion being induced. It was not repeated, and the child was born naturally. Similar instances have now and then been related as occurring in England. We regret, however, to have to say that in two cases lately reported in the Provincial Med. and Surg. Journal, a decidedly unfavorable result followed the use of the ether; in one the death of the patient is attributed to it wholly. The first case alleged is as follows: A man æt. 52, was operated on for stone; a little delay occurred in grasping the stone, but the whole operation lasted only ten minutes, during which the ether was administered at intervals. The breathing first became heavy and then stertorous: he recovered however from its effects after a short time, and then continued in a quiet, passive state, but without decided reaction, for twenty-four hours. At this period he had a severe chill: he then lay in a dozing state for some time, and then became completely collapsed or prostrated. He was stimulated all night, and next morning slight reaction occurred. He gradually sank again however, and died in the afternoon, being sensible to the last. There was membranous congestion of the brain, but no effusion; lungs anteriorly ex-sanguineous, posteriorly engorged, heart flaccid and empty, blood fluid throughout the whole vascular system. The post mortem was made 67 hours after death. The operator, Mr. Nunn, (Surgeon to the Colchester and Essex Hospital,) does not attribute the loss of his patient wholly to the influence of the ether, yet seems inclined to the opinion that it produced a very serious depressing effect on the nervous system. He queries whether a patient, after the administration of ether, has not a double shock to overcome—that produced by the vapor, superadded to that of the operation itself? and doubts whether the prevention of pain, which as he considers as a natural incentive, in its effects on the system, to reparative action, be so vital a desideratum in operative surgery."

"*Inquest.*—An inquest has been held on a young woman, the wife of a hair-dresser, at Spittlegate, Lincolnshire, from whom a tumor has been removed, while under the influence of ether. *She never rallied;* and died withont the slightest reaction having taken place, 10 hours after the operation. The following verdict was returned: "That the deceased died from the effects of the vapor of ether, inhaled by her for the purpose of alleviating pain during the removal of a tumor from her left thigh, and not from the effects of the operation, or from any other cause.

"The surgeon who performed the operation stated that he fully concurred in the verdict and had no doubt whatever that the ether alone was the cause of death, and it was a duty he owed the public to say so."—*Bost. Med. and Surg. Journal,*

"A boy had his arm badly crushed in Boston a few days since, and it was amputated by Dr. Lewis while the patient was under the influence of ether. In a few hours after he died. The ether was properly administered by Dr. Hayward, Surgeon of the Massachusetts Hospital, who, with others, was present at the operation.. It seems from the report, that in the opinion of Dr. Lewis, "the inhalation of the ether was the immediate cause of the boy's death." —*N. Y. Annalist.*

